# Four decades of developmental dysplastic hip screening according to Graf: What have we learned?

**DOI:** 10.3389/fped.2022.990806

**Published:** 2022-09-15

**Authors:** Sebastian G. Walter, Robert Ossendorff, Ayla Yagdiran, Jan Hockmann, Rahel Bornemann, Sonja Placzek

**Affiliations:** ^1^Department for Orthopedic Surgery and Traumatology, University Hospital Cologne, Cologne, Germany; ^2^Department for Orthopedic Surgery and Traumatology, University Hospital Bonn, Bonn, Germany; ^3^Medical Service of the Health Funds (MDK), Cologne, Germany

**Keywords:** Graf, DDH, hip, ultrasound, dysplasia

## Abstract

**Purpose:**

Sonographic hip examination according to Graf is widely accepted standard for diagnosing developmental dysplastic hips (DDH) but it is criticized for alleged intra- and interobserver variations. This review was conducted to evaluate whether objective quality criteria according to the Graf method are fulfilled within scientific literature.

**Methods:**

A systematic literature search on Pubmed was performed using the search string: [(DDH) OR (Hip dysplasia)] AND (Graf). Studies suitable by title, abstract, manuscript, containing an image of sonographic hip examination and online accessibility were included into analysis.

**Results:**

131 studies were included into final analysis. Only 68 (51.9%) presented correct sonographic images according Graf’s criteria. 98 (74.8%) studies plotted alpha-angles (angle between bony roof line and base line) but only 85 (64.8%) studies beta-angles (angle between cartilage roof line and base line). Studies were contributed from 25 countries.

**Conclusion:**

Assumingly, skepticism regarding the Graf method is based on user errors and insufficient application of the Graf quality assessment algorithm resulting in high intra- and interobserver variations. When performed correctly, the Graf method is of high diagnostic value.

## Introduction

It was in 1983 when Graf introduced his technique for examination of infantile hips by ultrasound and he was confronted with skepticism from the orthopedic community. Nevertheless, his scientific approach proved to be of high diagnostic value (1, 2). Notably, the Graf method prevailed in the scientific debate and clinical practice over other ultrasound examination protocols and modifications (3, 4). Subsequently the ultrasound examination according to Graf became standard within German-speaking countries and is nowadays part of a general newborn screening (5, 6). This method proved to be powerful to detect developmental dysplastic hips (DDH) at a very early age and is therefore associated with significantly reduced rates of severe dysplasia of adults in these populations. However, there is still reluctance regarding this method in various regions of the world. It is argued both, that the method shows high intra- and interobserver variabilities and that a general screening leads to an overtreatment (7–10). Although there are several studies rejecting the first argument, the second argument could not be verified by a Cochrane review, and both arguments are still common in oral debate (11–15).

Despite the fact that the Graf method is highly standardized, we considered the argument of inter-and intraobserver reliability respective inaccuracy of sonographic imaging according to the Graf criteria as reality-based experience from clinical work of practitioners and thus potentially hazardous for treatment decisions. It was hypothesized that reluctance to the Graf method correlates with inadequate sonographic figures in scientific literature.

## Materials and methods

**FIGURE 1:**
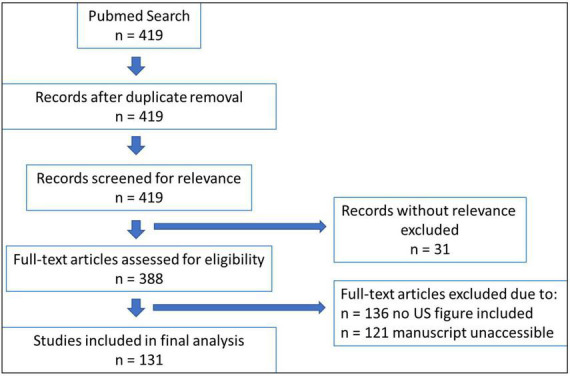
Study selection process according to the PRISMA scheme.

A systematic Pubmed search was performed using the Boolean Search String: “[(DDH) OR (Hip dysplasia)] AND (Graf)” resulting in a total of 418 studies found (December 2021). All studies were evaluated according to PRISMA criteria by title, abstract and by the original manuscript whether they were referring to the Graf method for diagnosis of DDH (Figure 1) (16). Inclusion criteria were complete manuscripts being accessible and presentation of exemplary figures of sonographic hip examinations. Studies were included independently of their language. Studies with figures referring explicitly to other techniques were excluded.

**FIGURE 2:**
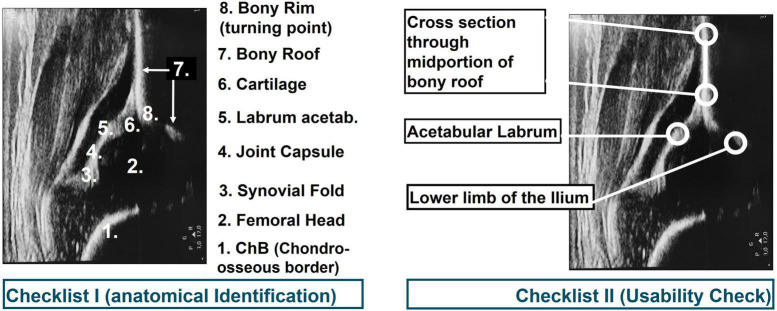
Checklist for correct (left) anatomical identification and (right) standard plane according to the Graf method.

All studies were reviewed independently by two orthopedic residents. Both had participated at the official Graf instruction course and had major experiences in infantile DDH diagnosis using the Graf method. Data were extracted from each study using a standardized spreadsheet. Evaluation criteria were correct fulfillment of quality criteria according to the Graf quality assessment algorithm (A: anatomical identification: 1. Chondro-osseous border, 2. Femoral Head, 3. Synovial fold, 4. Joint Capsule, 5. Labrum, 6. Cartilage, 7. Bony roof, 8. Bony rim (turning point); B: usability check: 1. Lower limb of the Ilium, 2. Cross section through mid-portion of the bony roof, and 3. Acetabular Labrum; Figure 2). Detected errors were documented as well as a (in-)correct marking of the alpha and beta angle. Furthermore, metadata of each study such as year of publication, country of first author, age of children at primary sonographic hip examination (if applicable), treatment method (if applicable), treatment success rate (if applicable) and type of study (original vs. review) were collected.

For all studies, two reviewers independently extracted information; differences were resolved where necessary with the help of a third person specialized in children’s orthopedics.

Publications presenting figures of congenital hip dislocation were rated as correct as the standard plane according to Graf is not achievable in these cases. Systematic user error was assumed when illustrations in manuscripts were presented as correct according to the Graf method but did not meet the quality criteria according to the Graf method.

Statistical heterogeneity among studies was not examined due to above described figurative endpoints.

## Results

The Pubmed Search resulted in 419 listed publications. Of these, 31 studies were excluded due to missing relevance. Another 135 studies did not contain a figure of a sonographic hip examination and for 121 studies the manuscript was not accessible online. 131 studies were included into final analysis for this review. Studies are listed by Pubmed-ID in Table 1. In 63 studies (48.1%) quality criteria according to Graf were not met. The majority of these studies insufficiently depicted anatomical landmarks. In 43 studies, pictures were presented without the possibility to identify all eight anatomical landmarks (e.g., images without a chondro-osseous border, cartilaginous roof not identified, joint capsule confused with intermuscular septum, etc.). 13 studies (9.9%) presented images that were not within the standard plane. A tilting error was detected in 7 studies (5.3%) only. The α-angle measurement was shown in 98 studies (74.8%), the β-angle was determined graphically in 86 studies (65.6%). We observed incorrectly plotted lines for the α-angle in 26 studies (19.8%). In most cases, the line through the *os ilium* was correct but the angulation line indicating the bony roof angle (α-angle) was plotted into the osseous parts of the acetabulum instead of running tangential beneath it.

**TABLE 1:** Pubmed-IDs (PMID) of all 131 analyzed studies.

PMID							
7458597	10036739	1613080	16596510	6666247	8571657	29080986	9481659
3512001	10549115	8469585	17929536	21567151	8781102	29524009	16601983
3324535	10868360	8211098	18300148	6652194	9064764	29125399	10067213
3303351	11386103	7941683	18493759	6712426	9214167	29509577	26052572
3316826	12510090	7946820	20048103	6392336	28246872	31534923	10904906
3275689	12089496	8543602	31305360	3906256	9304198	30483824	26432791
2057752	12754458	7883916	31503108	20408115	31634952	30630451	11253556
6874934	15602221	25476594	31882168	3534775	32503452	20440222	28497455
23205143	15076582	24674894	34100358	3016821	32356221	19829154	12029342
22144750	15091252	27847852	34258040	3296175	31385896	33652770	28736369
31528565	15703518	27489571	34405425	22498841	32434356	34008885	1437367
22410971	29194074	27084324		22579539	16365130	15812286	28717864
23752151	29727411	26092660		24286094	16697598	16093942	30564018
24619870	29688160	28341489		24811088	17235535	27151910	30154920
24964047	30996739	28025728		2190155	34641800	26047647	21184670
24590339	30327989	15995619		25116241	34189091	28405809	15611889
25561752	9683715	16638445	31344996	24510434	34128864	20544189	30526178

Studies marked yellow presented sonographic images that were insufficient to quality criteria according to the Graf method.

A large proportion of studies originated from Europe and German-speaking countries (Germany 29 studies; Austria 17 studies; Turkey 22 studies; Canada 8 studies; United States 7 studies; China 6 studies; United Kingdom and Brazil 5 studies each; Italy and Netherlands 4 studies each; Romania 3 studies; Croatia, France, Japan, Norway, Poland, Switzerland, Taiwan 2 studies each; Denmark, Egypt, Iran, Mongolia, Serbia, Singapore, Sweden 1 study each). German-speaking countries had a significantly higher rate (*p* = 0.02) of correct sonographic images than other countries (percentages of correct sonographic images: Germany: 62.1%, Austria: 70.6%, Turkey: 54.5%, United Kingdom: 40.0%, United States: 14.3%, Brazil: 20%, China: 66.7%, Netherlands: 50.0%, Italy: 50.0%, Canada: 0%).

There were 14 review articles (10.7%) and 117 original works (89.3%). 105 studies (80.1%) contributed data to the age of children at sonographic hip examination. In all but 10 studies the age was less than 3 months. Among the 10 studies reporting on children more than 6 months of age at sonographic hip examination, there were cases of initial failed therapy, delayed diagnosed included accounting for late (mostly secondary) examination. Maximum ages reported for sonographic hip examination were 83 weeks respective 5 years.

## Discussion

Hip dysplasia represents a pre-arthritic deformity and it is estimated that 10–15% of patients requiring hip arthroplasty beneath the age of 50 do so because of dysplastic hips (17, 18). Without doubt the introduction of standardized hip sonography by Graf in the early 1980s has significantly contributed to fewer cases of severe hip dysplasia in adults requiring extensive surgery (5, 19).

Although universal infantile hip screening programs in German-speaking countries have shown to be very successful, there is still significant reluctance regarding this established method. Overtreatment in DDH therapy is suspected (20). Yet, it is essential to differentiate between different degrees of DDH. While IIa hips according to Graf may mature into type I hips without additional treatment, it is general consent that Graf type III and IV hips need to be treated to prevent severe dysplasia in adults and identification of these hips is based on clinical examination and sonographic imaging according to the Graf method. Nevertheless, there are authors claiming, that those hips will turn healthy after some months of follow-up, basing their conclusion on sparse data (21). Principally, dislocated or instable hips (Graf type III or IV) can be treated either by closed reduction or by Pavlik harness (22, 23). Closed reduction yields the advantage of immediate reposition control (24).

Although the Graf method is the most known and widespread technique for sonographic examination of pediatric hips, there is partial dissent on which classification or sonographic method is to be used for diagnosis of DDH. So far, no study demonstrated superiority regarding specifity and sensitivity of other sonographic examination protocols over the Graf method. In contrast, there is evidence indicating that criteria according to Graf (α-angle) are better reproducible than femoral head coverage as used in the Terjesen classification (3). Furthermore, a study by Roposch and colleagues described in an illustrative manner the controversies among orthopedic surgeons from different regions and countries (25).

This current review aims to identify reasons why the standardized method according to Graf is still matter of debate regarding reproducibility and practicability. Although authors within analyzed studies (Tab. 1) referred to the Graf method, a large percentage (48.1%) of these studies did not fulfill basic criteria for performing the Graf method. Therefore, it becomes evident that the term Graf is widely known but the underlying method is not applied correctly in the scientific and subsequently the clinician community that orientates on recent advances and publications in this field. Hence, this might explain why the Graf method remains subject to criticism regarding intra- and interrater variability.

A higher rate of correct images was found in publications originating from German-speaking countries, which might be explained by a higher density of trainings on this method and the integration of this method into an obligatory pediatric screening programs. Unfortunately, the rate of incorrect sonographic images in scientific publications per year did not decrease since the introduction of the Graf method.

It has to be admitted that this review evaluated only representative images in analyzed publications and may thus be subject to bias. However, authors are keen to select most representative and accurate images to demonstrate their measurement and method of data acquisition in order to support their findings and scientific theory. If those images are erroneous, it is justifiable to conclude that a majority of other measurements are incorrect as well. Yet, it is highly questionable if every incorrect image led to an incorrect treatment.

A large percentage of initially detected publications was not accessible online and thus excluded from final analysis. This may bias the results of this study. However, this problem of limited access to certain original manuscripts is a general problem for all researchers and clinicians researching on DDH. These online inaccessible manuscripts cannot portray correct or incorrect images to the community and therefore cannot not change the overall impression of inadequate application of the Graf method.

**FIGURE 3:**
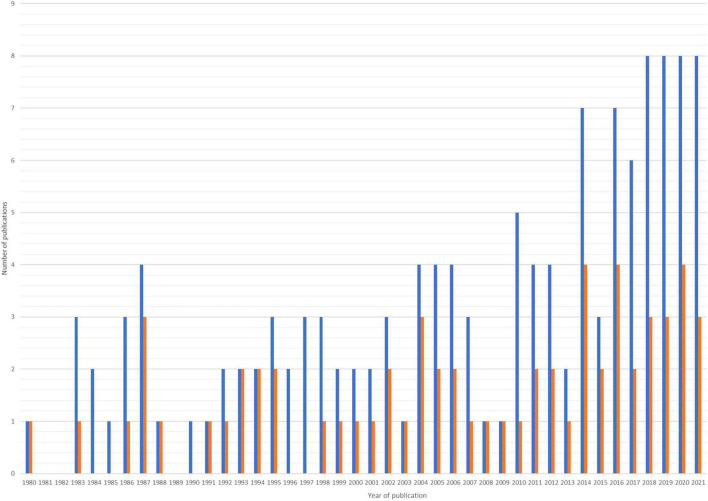
Publications by year. Blue are publications in a certain year and orange are publications with incorrect sonographic images according to the Graf method. Only publications that were analyzed in this review were included in this chart.

One study published by Graf himself in 1980 was included in this review for historical reasons, although the Graf method was finally introduced in 1983 (Figure 3). For reasons of publishing rights and collegiality no examples from analyzed publications were shown in this manuscript.

In conclusion, the Graf method is known for four decades but there are still major reservations and skepticism among clinicians and scientists around the globe regarding this method, which may be partly based on incorrect sonographic images in scientific publications. When performed correctly, however, the Graf method is of high diagnostic value.

## Author contributions

SP and SW contributed to the study conception and design. SW wrote the first draft of the manuscript. All authors performed the material preparation, data collection and analysis, commented on previous versions of the manuscript, and read and approved the final manuscript.
